# MYSM1-AR complex-mediated repression of Akt/c-Raf/GSK-3β signaling impedes castration-resistant prostate cancer growth

**DOI:** 10.18632/aging.102482

**Published:** 2019-11-24

**Authors:** Jinbo Sun, Xiangnan Hu, Yongheng Gao, Qisheng Tang, Zhining Zhao, Wenjin Xi, Fan Yang, Wei Zhang, Yue Song, Bin Song, Tao Wang, He Wang

**Affiliations:** 1 Department of Urology, Tangdu Hospital, Fourth Military Medical University, Xi'an, Shaanxi 710038, China; 2 Department of Respiratory and Critical Care Medicine, Tangdu Hospital, Fourth Military Medical University, Xi'an, Shaanxi 710038, China; 3 Clinical Laboratory, 451 Hospital of Chinese People's Liberation Army, Xi'an, Shaanxi 710054, China; 4 State Key Laboratory of Cancer Biology, Department of Immunology, Fourth Military Medical University, Xi’an, Shaanxi 710032, China; 5 Department of Orthopedics, Xijing Hospital, Fourth Military Medical University, Xi’an, Shaanxi 710032, China; 6 Department of Medical Genetics and Developmental Biology, Fourth Military Medical University, Xi’an, Shaanxi 710032, China

**Keywords:** castration-resistant prostate cancer, growth, MYSM1, AR, Akt

## Abstract

Epigenetic alterations that lead to dysregulated gene expression in the progression of castration-resistant prostate cancer (CRPC) remain elusive. Here, we investigated the role of histone deubiquitinase MYSM1 in the pathogenesis of prostate cancer (PCa). Tissues and public datasets of PCa were evaluated for MYSM1 levels. We explored the effects of MYSM1 on cell proliferation, senescence and viability both *in vitro* and *in vivo*. Integrative database analyses and co-immunoprecipitation assays were performed to elucidate genomic association of MYSM1 and MYSM1-involved biological interaction network in PCa. We observed that MYSM1 were downregulated in CRPC compared to localized prostate tumors. Knockdown of MYSM1 promoted cell proliferation and suppressed senescence of CRPC cells under condition of androgen ablation. MYSM1 downregulation enhanced the tumorigenic ability in nude mice. Integrative bioinformatic analyses of the significantly associated genes with MYSM1 revealed MYSM1-correlated pathways, providing substantial clues as to the role of MYSM1 in PCa. MYSM1 was able to bind to androgen receptor instead of increasing its expression and knockdown of MYSM1 resulted in activation of Akt/c-Raf/GSK-3β signaling. Together, our findings indicate that MYSM1 is pivotal in CRPC pathogenesis and may be established as a potential target for future treatment.

## INTRODUCTION

Prostate cancer (PCa) is one leading cause of cancer-related death among men in Western countries [1]. Although surgical intervention has been shown to be efficacious in eradicating localized PCa, androgen deprivation therapy (ADT), aimed at suppressing androgen synthesis or androgen receptor (AR) activity [2, 3], remain the mainstay treatment for locally advanced or metastatic PCa. Unfortunately, the majority of patients who have undergone ADT, even initially effective in tumor regression, eventually become resistant to the treatment [4] and progress to be castration-resistant PCa (CRPC). Castration resistance represents an enormous clinical challenge and improvements in therapies as well as causative biomarkers for CRPC are in urgent need.

AR with transcription activity is expressed in CRPC and may play an important role in the setting of castration via intratumoral androgen biosynthesis or interplay with other growth-promoting and prosurvival pathways [5, 6]. The heterogeneity in AR expression or activity suggest that AR deficiency may be proposed as a potential way in which prostate cancer cells escape androgen deprivation therapy [7], with compensatory signaling pathways activated concomitantly. The PI3K/Akt pathway is a well-established oncogenic pathway in human cancer [8–10] and is involved in resistance to AR-targeted therapy in prostate cancer [11–13]. Alterations of nodes in PI3K/Akt pathway are reported to occur in 42% of primary PCa and 70% of metastatic tumors [14]. PTEN loss is frequently observed during PCa progression, especially in advanced prostate tumors [15–17]. Thus, the PI3K/Akt pathway, which is antagonized by tumor suppressor PTEN, is constitutively activated in prostate cancers with PTEN deficiency, leading to enhanced tumor cell survival, metastasis and castration-resistant growth [12, 18, 19]. In addition, reciprocal feedback regulation between PI3K/Akt and AR signaling has been identified as a potent mechanism of CRPC progression and a crucial issue for monotherapies targeting AR or PI3K/Akt pathways [20–22]. Combination therapy cotargeting PI3K/Akt and AR signaling leads to significant regression of prostate cancer when compared with monotherapies [23–25], suggesting a coordinative role in supporting tumor survival.

Myb-like SWIRM and MPN domains 1 (MYSM1) acts as a histone H2A deubiquitinase and is responsible for removal of ubiquitin from monoubiquitinated histone H2A at lysine 119 (H2AK119ub) [26]. Early studies have linked MYSM1 to hematopoiesis where MYSM1 modulates hematopoietic stem cell function and survival [27–29]. By coordinating histone modifications and transcription factors recruitment, MYSM1 plays a critical role in control of lymphocyte differentiation and tissue development [29, 30]. Additionally, it has been reported that MYSM1 functions as a central negative regulator of inflammatory response and immune system to prevent excessive inflammation and self-destructive immune response [31, 32]. Little is known about the role of MYSM1 in human cancers. Few studies found that MYSM1 was involved in melanoma growth and colorectal cancer metastasis [33, 34]. It is worth noting that MYSM1 was reported to participate in regulation of AR-dependent gene transcription in PTEN-deficient LNCaP cells [26]. However, the prostate-specific role of MYSM1, particularly its role in CRPC, has not been explored in detail.

In this study, we investigated the role of MYSM1 in carcinogenesis and progression of PCa. We revealed the decreased expression of MYSM1 and confirmed its tumor-suppressing functions in CRPC. Mechanistically, MYSM1 may exert its effect through interplay with AR and inhibiting the activation of PI3K/Akt signaling. This work suggests that MYSM1 can be functionally essential for CRPC progression.

## RESULTS

### MYSM1 is downregulated in castration-resistant prostate cancer and inversely correlated with progression of prostate cancer

**Figure 1:**
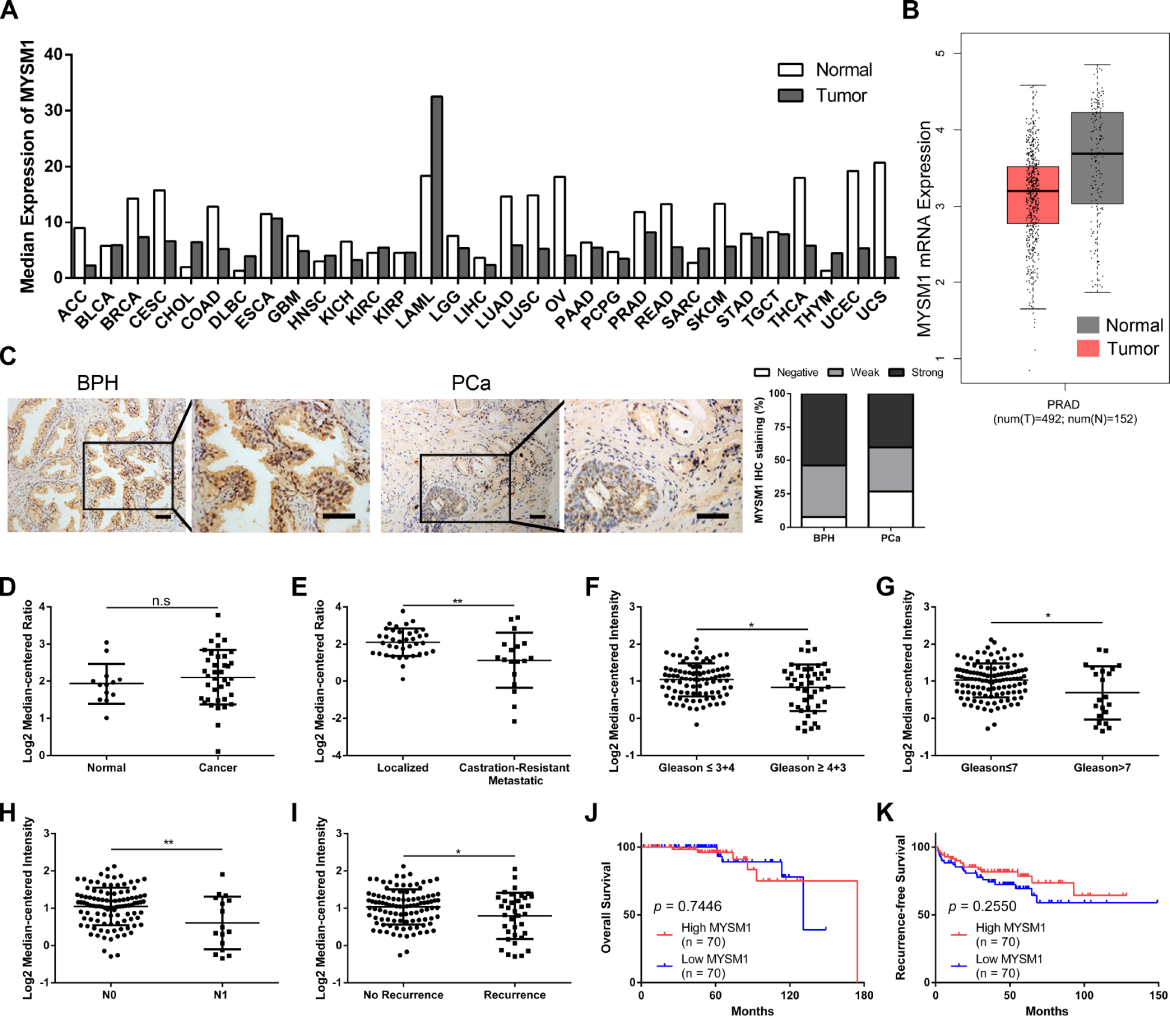
**MYSM1 is downregulated in castration-resistant prostate cancer and inversely correlated with progression of prostate cancer.** (**A**) Pan-cancer analyses for mean expression levels of MYSM1 in different types of cancers. (**B**) MYSM1 mRNA levels in prostate cancers (T = tumor, N = normal, PRAD = prostate adenocarcinoma). Data (**A**–**B**) were acquired from TCGA database and analyzed via GEPIA bioinformatics. (**C**) Representative IHC staining images of MYSM1 in benign prostatic hyperplasia (BPH) and prostate cancer (PCa) tissues. Scale bars are 20 μm. (**D**–**E**) MYSM1 expression levels based on Grasso Prostate dataset from Oncomine database. (**F**–**I**) MYSM1 is differentially expressed in prostate cancer patients and data acquired from Taylor Prostate 3 cohort are analyzed based on Gleason grade (**F**), Gleason score (**G**), N stage (**H**) and recurrence status (**I**) via Oncomine database. Data are shown as mean ± SD. n.s = no significance, * P < 0.05 and ** P < 0.01 (Student’s t-test). (**J**–**K**) Correlation of MYSM1 with overall survival (**J**) and recurrence-free survival (**K**) in prostate cancer patients (n = 140). Data were acquired from Taylor Prostate 3 cohort via Oncomine database. Log-rank test was applied to determine the significance levels.

To explore the expression of MYSM1 in human tumors, we performed web-based data mining to analyze The Cancer Genome Atlas (TCGA) datasets via GEPIA bioinformatics. We found that MYSM1 mRNA levels were downregulated in ACC (adrenocortical carcinoma), CESC (cervical squamous cell carcinoma and endocervical adenocarcinoma), COAD (colon adenocarcinoma), LUAD (lung adenocarcinoma), LUSC (lung squamous cell carcinoma), OV (ovarian serous cystadenocarcinoma), READ (rectum adenocarcinoma), SKCM (skin cutaneous melanoma), THCA (thyroid carcinoma), UCEC (uterine corpus endometrial carcinoma) and UCS (uterine carcinosarcoma) compared with normal tissues (Figure 1A, Supplementary Figure 1). In addition, MYSM1 expression was higher only in DLBC (lymphoid neoplasm diffuse large B-cell lymphoma) and THYM (thymoma) than that in normal tissues (Figure 1A, Supplementary Figure 2). The expression of MYSM1 was decreased in PRAD (prostate adenocarcinoma) compared with that in normal prostate glands, but the change was not statistically significant (Figure 1A, 1B). Immunostaining analyses revealed that MYSM1 protein levels were a little lower in prostate cancers when compared with benign prostatic hyperplasia (Figure 1C).

To further verify the expression of MYSM1 in prostate cancer, we analyzed 2 microarray datasets (Grasso Prostate and Taylor Prostate 3) from Oncomine database. Similarly, no significant differences of MYSM1 levels were observed between prostate cancers and prostate glands (Figure 1D). However, we found that compared with localized PCa patients, the expression of MYSM1 was significantly downregulated in metastatic castration-resistant prostate cancer (CRPC) patients (Figure 1E). Moreover, Oncomine analyses also revealed the clinically relevant signatures of MYSM1 expression in human PCa progression (Table 1). Although MYSM1 expression did not significantly correlate with age, PSA level, T stage, extracapsular extension, seminal vesicle involvement, surgical margins status, hormone therapy, chemotherapy and radiotherapy (Table 1, *P* > 0.05), the level of MYSM1 was inversely associated with Gleason grade, Gleason score, N stage and recurrence status (Figure 1F–1I). Kaplan-Meier analyses of Taylor Prostate 3 cohort indicated no significant associations of MYSM1 expression with overall survival and recurrence-free survival in PCa patients (Figure 1J, 1K). The reason for insignificant correlation of MYSM1 mRNA level with prognosis may be due in part to insufficient sample size. Taken together, these results suggest that dysregulation of MYSM1 may play a significant role in PCa progression and contribute to development of castration resistance.

**Figure 2:**
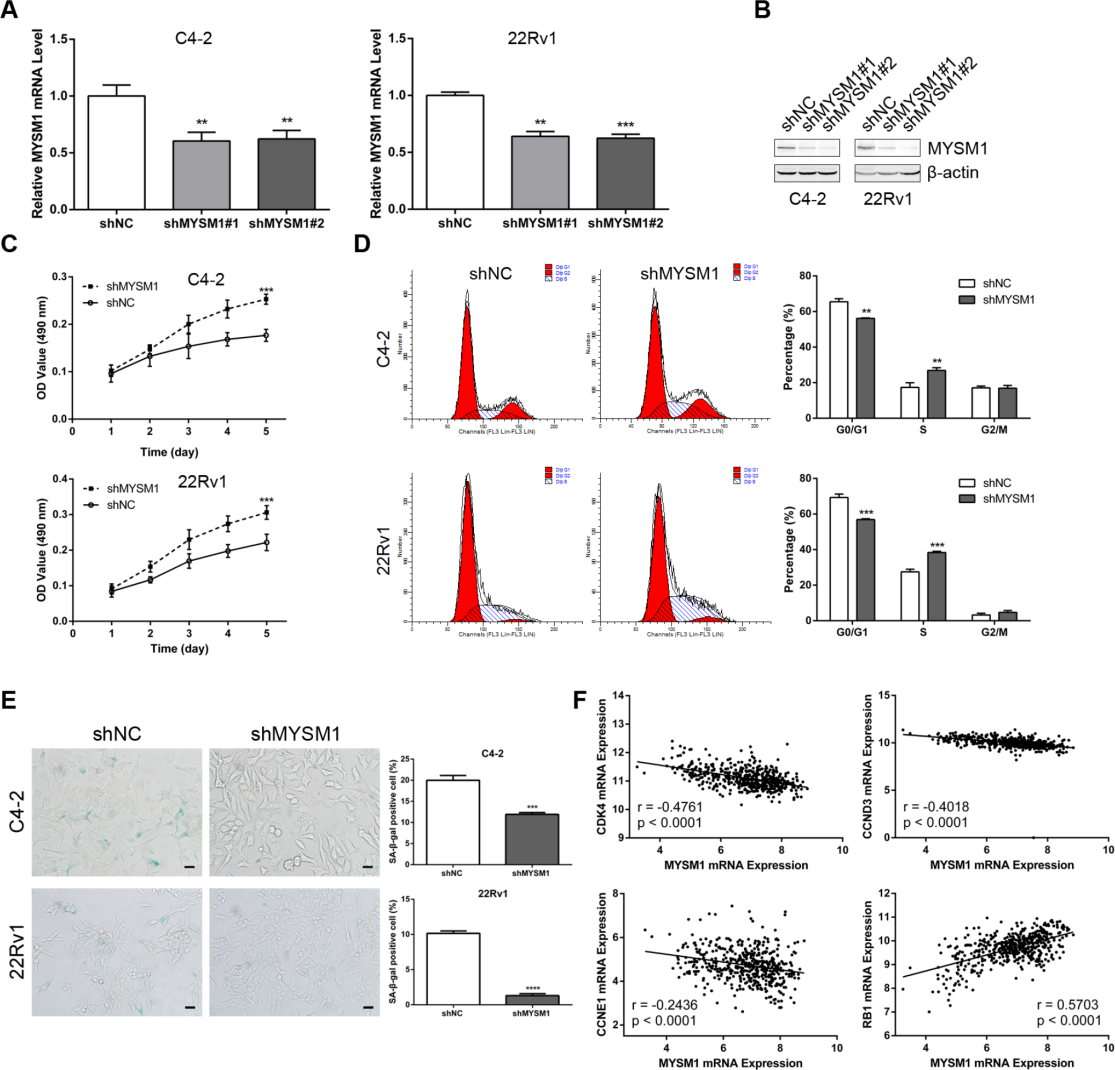
**MYSM1 knockdown promotes proliferation and suppresses senescence of prostate cancer cells.** (**A**–**B**) MYSM1 expression levels in CRPC cell lines (C4-2, 22Rv1) stably expressing shRNA targeting MYSM1 (shMYSM1) or negative control (shNC) were detected by qRT-PCR (**A**) and Western blot (**B**) analyses. Data are shown as mean ± SEM of 3 replicates. ** P < 0.01 and *** P < 0.001 (one-way ANOVA test). (**C**) Proliferation of shNC/shMYSM1-treated C4-2/22Rv1 cells was evaluated by MTT assay. Data are shown as mean ± SEM of 3 replicates. (**D**) Flow cytometry analysis of cell cycle in C4-2/22Rv1 cells treated with shNC/shMYSM1. Data are shown as mean ± SD of 3 replicates. ** P < 0.01 and *** P < 0.001 (Student’s t-test). (**E**) Representative images of SA-β-gal staining in C4-2/22Rv1 cells treated with shNC/shMYSM1. Scale bars are 25 μm. Data are shown as mean ± SD of 3 replicates. *** P < 0.001 and **** P < 0.0001 (Student’s t-test). (**F**) Correlation of MYSM1 with CDK4, CCND3, CCNE1 and RB1 mRNA levels in prostate cancers. Data were collected from TCGA database and analyzed via LinkedOmics bioinformatics. Pearson correlation coefficients and significance levels are indicated.

**Table 1:** Demographic and Clinicopathological Characteristics of Prostate Cancer Patients (Taylor Prostate 3 Cohort) and Association of MYSM1 Expression with Clinicopathological Parameters (Chi-square test).

**Clinicopathological parameters**	**Frequency (%)**	**MYSM1 mRNA expression**		**P value**
		**< median**	**≥median**	
Age (n=150)				
< 60	93 (62.0)	43	50	0.239
≥ 60	57 (38.0)	32	25	
Pre-diagnosis biopsy PSA (n=147)				
< 10 ng/ml	115 (78.2)	59	56	0.450
≥ 10 ng/ml	32 (21.8)	14	18	
Pre-treatment PSA (n=147)				
< 10 ng/ml	105 (71.4)	55	50	0.297
≥ 10 ng/ml	42 (28.6)	18	24	
T stage (n=141)				
T1-T2	86 (61.0)	40	46	0.352
T3-T4	55 (39.0)	30	25	
Extracapsular extension (n=141)				
None	43 (30.5)	19	24	0.361^a^
Capsular invasion	47 (33.3)	23	24	
Focal	7 (5.0)	2	5	
Established	44 (31.2)	26	18	
Seminal vesicle involvement (n=141)				
Negative	119 (84.4)	58	61	0.617
Positive	22 (15.6)	12	10	
Surgical margins (n=141)				
Negative	108 (76.6)	51	57	0.298
Positive	33 (23.4)	19	14	
Hormone therapy (n=150)				
No	115 (76.7)	55	60	0.334
Yes	35 (23.3)	20	15	
Chemotherapy (n=150)				
No	136 (90.7)	65	71	0.092
Yes	14 (9.3)	10	4	
Radiotherapy (n=150)				
No	126 (84.0)	62	64	0.656
Yes	24 (16.0)	13	11	

^a^Fisher exact test.

### MYSM1 knockdown promotes proliferation and suppresses senescence of CRPC cells

The observation that the MYSM1 expression decreases as the tumor develops castration resistance led us to evaluate the role of MYSM1 in CRPC. To determine the putative function of MYSM1 in castration-resistant growth of PCa, we downregulated MYSM1 levels in androgen-independent C4-2 and 22Rv1 cell lines using lentivirus MYSM1 shRNAs (shMYSM1) and negative control shRNA (shNC). The efficient knockdown of MYSM1 was confirmed by measuring MYSM1 expression at the mRNA (Figure 2A) and protein (Figure 2B) levels. Cells were cultivated in RPMI-1640 medium supplemented with charcoal-stripped serum to mimic the hormone-starvation conditions. MTT and flow cytometry assays were performed to investigate the influence of MYSM1 knockdown on proliferation and cell cycle in C4-2 and 22Rv1 cells stably expressing shRNA targeting MYSM1 or negative control. We found that MYSM1 silencing in CRPC cells significantly increased the proliferation as shown in MTT assays (Figure 2C). Similarly, a significant change of cell cycle distribution was detected in MYSM1-deficient cells. There was a decrease in the percentage of G1-phase cells and an increase in that of S-phase cells (Figure 2D), indicating an accelerated progression of cell cycle. Because it has been reported that promoted cell growth and cell cycle progression might be mediated by suppression of senescence and apoptosis, we next assessed whether MYSM1 deletion in CRPC cells would inhibit senescence and apoptosis induction. Therefore, we performed SA-β-gal staining assays to evaluate cellular senescence phenotype. Our results showed that MYSM1 downregulation led to decreased proportion of β-gal positive cells (Figure 2E). Moreover, cells transfected with siRNAs against MYSM1 and NC for 48h were subjected to apoptosis analysis via Annexin V/PI-labeling flow cytometry. However, our results showed that MYSM1 depletion did not exert considerable influence on apoptosis in CRPC cells (Supplementary Figure 3). Resistance to senescence or apoptosis has been proposed as a strategy for cancer cell survival and tumor growth promotion. In agreement with our results, prior research has shown that some cells are more susceptible to senescence rather than apoptosis even after undergoing extensive extrinsic stimuli [35]. Further analyses of PCa patients from LinkedOmics database revealed a negative correlation between MYSM1 mRNA and gene transcripts related to proliferation and cell cycle, including CDK4, CCND3 and CCNE1 (Figure 2F). Moreover, we observed that MYSM1 transcription was strongly associated with the expression of tumor suppressor RB1 in PCa patients (Figure 2F). Collectively, these data indicate that MYSM1 expression in CRPC cells results in the suppression of androgen-independent growth and induction of cell cycle arrest as well as cellular senescence, suggesting a tumor-suppressive role of MYSM1 in CRPC cells.

### Downregulation of MYSM1 facilitates prostate cancer growth *in vivo*

**Figure 3:**
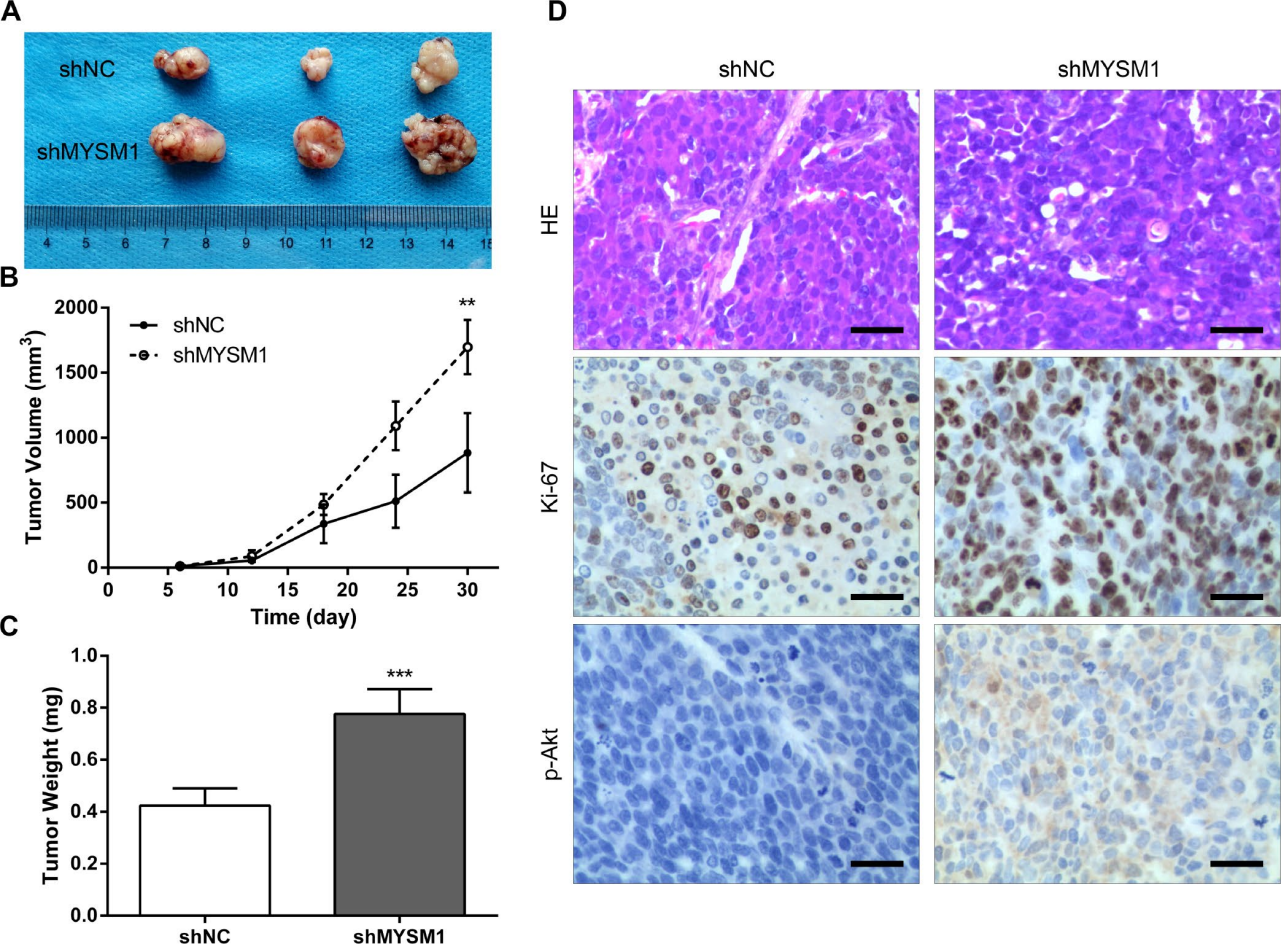
**Downregulation of MYSM1 facilitates prostate cancer growth in vivo.** (**A**) Representative image of tumors formed in nude mice bearing 22Rv1/shNC or 22Rv1/shMYSM1 cells. (**B**) The volumes of tumors derived from nude mice subcutaneously implanted with 22Rv1/shNC or 22Rv1/shMYSM1 cells were monitored for 30 days. Data are shown as mean ± SD of 3 replicates. (**C**) The animals were sacrificed 30 days after injection and tumor weights were evaluated. Data are shown as mean ± SD of 3 replicates. (**D**) Representative photographs of IHC staining of p-Akt and Ki-67 in tumor tissues from 22Rv1/shNC and 22Rv1/shMYSM1 nude mice groups. Scale bars are 30 μm. ** P < 0.01 and *** P < 0.001 (Student’s t-test).

To further determine the anti-tumor role of MYSM1 in CRPC growth *in vivo*, we subcutaneously engrafted nude mice with 22Rv1 cells stably expressing shRNA targeting either MYSM1 or negative control and tracked tumor growth. Our results showed that the tumors formed in mice from 22Rv1/shMYSM1 group were significantly larger than those from 22Rv1/shNC group (Figure 3A). Moreover, we observed that the inhibition of MYSM1 significantly affected tumor growth in nude mice (Figure 3B). In addition, tumor weights of 22Rv1 xenografts in immunodeficient mice were significantly increased by downregulating MYSM1 levels (Figure 3C). To further confirm the functional impact of MYSM1 on tumor growth, we performed IHC staining to analyze tumor tissues from nude mice. Our results showed that the protein level of Ki-67, a marker for proliferation, was significantly upregulated in tumor sections from the 22Rv1/shMYSM1 group compared with those from the 22Rv1/shNC group (Figure 3D). Consistently, IHC staining for p-Akt, constitutively activated in CRPC cells, showed that downregulation of MYSM1 resulted in a significant increase in p-Akt expression in tumor tissues derived from mice implanted with 22Rv1/shMYSM1 cells (Figure 3D). Together, these data indicate that MYSM1 negatively regulates CRPC growth *in vivo*.

### Integrative multi-omics analysis of MYSM1 mRNA expression in prostate cancers

**Figure 4:**
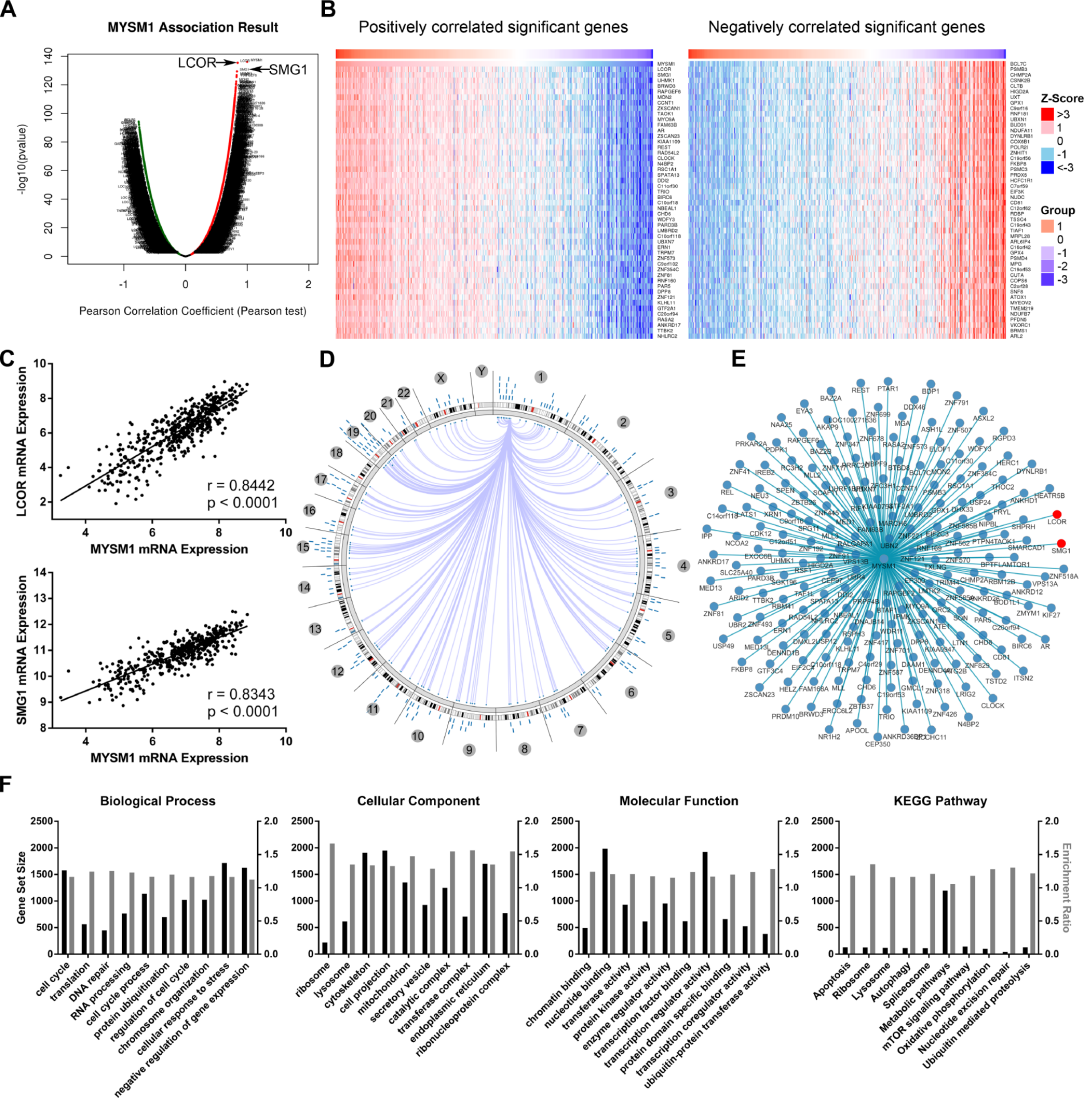
**Integrative multiomics analysis of MYSM1 mRNA expression in prostate cancers.** (**A**) Association of MYSM1 with genes differentially expressed in prostate cancers. (**B**) Heatmaps for genes positively (left) and negatively (right) correlated with MYSM1 in prostate cancers (top 50). (**C**) Correlation of MYSM1 with LCOR (upper) and SMG1 (lower) mRNA levels in prostate cancers. Pearson correlation coefficients and significance levels are indicated. Data (**A**–**C**) were collected from TCGA database and analyzed via LinkedOmics bioinformatics. (**D**) Statistically significant associations of MYSM1 with genomic coordinates are indicated by arcs connecting pairs of dots in prostate cancers. (**E**) Network view of correlations between MYSM1 and other genes in prostate cancers. Data (**D**–**E**) were acquired from TCGA database and analyzed via Regulome Explorer. (**F**) Significantly enriched GO annotations and KEGG pathways of genes correlated with MYSM1 in prostate cancers. Data were acquired from TCGA database and analyzed via GSEA bioinformatics.

The LinkFinder module of LinkedOmics database was used to explore the association between MYSM1 mRNA and other gene expression by analyzing the RNA sequencing data from TCGA PCa patient cohort (n = 497). As indicated by the volcano plot (Figure 4A), 6624 genes (dark red dots) displayed significant positive correlation with MYSM1 expression, whereas 6088 genes (dark green dots) showed strong negative association (FDR < 0.01). The top 50 most significant genes positively and negatively associated with MYSM1 mRNA level were visualized in the heat maps (Figure 4B). These findings suggest a widespread influence of MYSM1 on the transcriptome. The LinkFinder also output statistical scatter plots for individual genes. MYSM1 gene transcript level demonstrated a strong positive association with the expression of LCOR (positive rank #1, *r* = 0.84, *P* = 3.94e-136) and SMG1 (positive rank #2, *r* = 0.83, *P* = 4.30e-130) (Figure 4C). Both LCOR and SMG1 protein are known to function as a tumor suppressor in multiple cancers and mediate the repression of tumor growth by regulating oncogenic proteins involved in cell cycle [36, 37]. Using Cancer Regulome tools, further analyses of TCGA PCa patients were performed to explore the genome-level associations of MYSM1 expression with various molecular features, including gene expression, DNA methylation, somatic copy number, microRNA expression, somatic mutation and protein level (RPPA). The significant associations between MYSM1 level and gene expression features within the context of genomic coordinates were presented as a circular graph (Figure 4D) and a network (Figure 4E). Consistent with the results in Figure 4A–4C, the transcription levels of LCOR and SMG1 (red) were significantly correlated with MYSM1 mRNA expression (Figure 4E). We next performed gene ontology (GO) and KEGG pathway analyses of genes significantly associated with MYSM1 expression using WebGestalt via LinkedOmics bioinformatics (Figure 4F). Our results indicated that the proteins encoded by these genes are localized mainly in cytoskeleton, cell projection and endoplasmic reticulum (FDR < 0.0001). They are significantly enriched in cellular response to stress, cell cycle and negative regulation of gene expression (FDR < 0.0001) and participate primarily in nucleotide binding, transcription regulator activity and enzyme regulator activity (FDR < 0.0001). KEGG pathway analysis revealed that MYSM1 mRNA was highly associated with genes involved in metabolic pathways and mTOR signaling pathway (FDR < 0.01). Taken together, these findings suggest a potential role of MYSM1 in the biological interaction network correlated with tumor progression.

### MYSM1 interacts with AR and inhibits activation of Akt/c-Raf/GSK-3β signaling in prostate cancer

**Figure 5:**
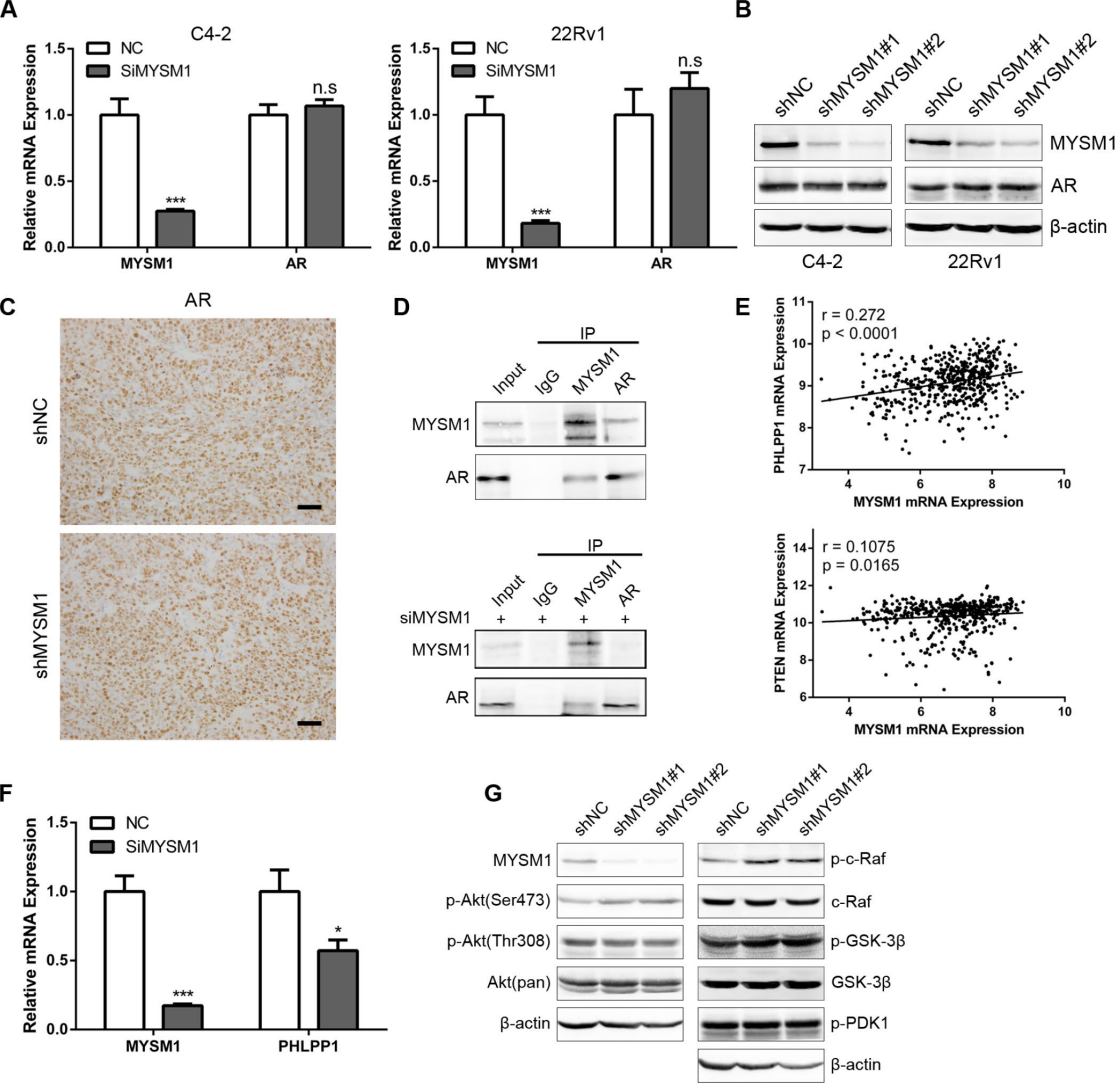
**MYSM1 interacts with AR and inhibits activation of Akt/c-Raf/GSK-3β signaling in prostate cancer.** (**A**) qRT-PCR analyses for MYSM1 and AR mRNA levels following transfection of NC/siMYSM1 into C4-2 and 22Rv1 cells. Data are shown as mean ± SEM of 3 replicates. (**B**) Western blot analyses for MYSM1 and AR protein levels in C4-2 and 22Rv1 cells treated with shNC/shMYSM1. (**C**) Representative IHC staining images of AR in xenograft tumor tissues. Scale bars are 30 μm. (**D**) Co-IP assays of MYSM1 and AR in C4-2 cells with (down) or without (up) siMYSM1 treatment. (**E**) Correlation of MYSM1 with PHLPP1 (up) and PTEN (down) mRNA levels in prostate cancers. Pearson correlation coefficients and significance levels are indicated. Data were collected from TCGA database and analyzed via LinkedOmics bioinformatics. (**F**) qRT-PCR analyses for MYSM1 and PHLPP1 mRNA levels following transfection of NC/siMYSM1 into C4-2 cells. Data are shown as mean ± SEM of 3 replicates. (**G**) Western blot analyses of lysates from C4-2 cells treated with shNC/shMYSM1 were probed with the indicated antibodies. n.s = no significance, * P < 0.05 and *** P < 0.001 (Student’s t-test).

Considering the significant role of MYSM1 in CRPC, we next sought to elucidate the molecular mechanisms by which MYSM1 suppressed CRPC progression. Previous studies have shown that LCOR can act as a corepressor for AR signaling [36] which is still indispensable for CRPC. As our results demonstrated a strong association between MYSM1 and LCOR, we thus focused on investigating whether MYSM1 regulates AR signaling. To study the effect of MYSM1 on AR expression, qRT-PCR (Figure 5A) and western blot (Figure 5B) analyses were performed to detect the expression levels of AR in MYSM1-downregulated CRPC cells. In addition, we performed IHC staining to analyze AR expression in xenograft tumor tissues from nude mice (Figure 5C). However, we observed that MYSM1 knockdown led to no apparent changes in AR expression (Figure 5A–5C). Given the repression of AR transactivation by interaction of LCOR with AR [36], we then concentrated on the interaction between MYSM1 and AR to dissect the functional significance of MYSM1 in AR signaling. To verify the potential interaction between MYSM1 and AR, we carried out protein co-immunoprecipitation (Co-IP) assays with validated antibodies against MYSM1 and AR in C4-2 cells with or without siMYSM1 treatment. The Co-IP results confirmed the binding between MYSM1 and AR in CRPC cells (Figure 5D), which may be conducive to the activation of AR signaling. A reciprocal feedback has been reported to exist between AR and Akt signal pathways [21, 22]. Bioinformatics analyses of a TCGA PCa cohort via LinkedOmics indicted that MYSM1 significantly correlates with the expression levels of PHLLP1 and PTEN (Figure 5E). Both PTEN and PHLPP1 are known to be key regulators in activation of Akt signaling. In addition, KEGG pathway analysis also revealed a significant enrichment of MYSM1-associated genes in mTOR signaling (Figure 4F). To further validate the link between MYSM1 and activation of Akt pathway, we performed qRT-PCR and western blot assays in C4-2 cells. The qRT-PCR analyses showed that siRNA-mediated downregulation of MYSM1 resulted in a significant decrease in PHLPP1 expression, confirming the association of MYSM1 with PHLPP1 (Figure 5F). Western blot results further indicated that stable knockdown of MYSM1 led to increased levels of p-Akt(S473), p-c-Raf and p-GSK-3β without affecting the expression of p-Akt(T308), p-PDK1 and total proteins including Akt, c-Raf and GSK-3β (Figure 5G). In summary, these findings demonstrate that MYSM1 can bind to AR and suppress the activation of Akt pathway in CRPC by downregulating phosphorylation levels of Akt, c-Raf and GSK-3β.

## DISCUSSION

In the present study, we identified the dynamic change of MYSM1 expression during PCa transformation and progression. We found that MYSM1 levels are downregulated in CRPC tissues compared to localized primary tumor tissues, indicating a potential role for MYSM1 in the switch to castration resistance. A series of functional assays demonstrated that MYSM1 is involved in proliferation and senescence of CRPC cells. Further mechanistic investigation indicated that MYSM1-AR interaction and Akt/c-Raf/GSK-3β pathway may underlie the role of MYSM1 in CRPC progression.

MYSM1 catalyzes deubiquitination of monoubiquitinated histone H2A at lysine 119 (H2AK119ub) [26]. A growing bank of evidence indicates that dysregulated H2AK119ub levels, which are modulated by various deubiquitinases and ubiquitin ligases, are involved in gene transcription [26, 38], DNA damage repair [39] and replication fork regulation [40], all of which could result in genomic instability and tumorigenesis. Furthermore, H2A deubiquitinases, such as BAP1, USP14 and USP28, have been shown to suppress proliferation and increase radiosensitization in human cancers [41–43]. In contrast, previous studies have revealed that ubiquitin ligases, such as RNF2 and BMI1, play a pivotal role in tumorigenesis and metastasis [44–46]. As a member of deubiquitinase family, MYSM1 has been reported to be associated with tumor progression in melanoma and colorectal cancer by only few studies with small sample sizes [33, 34]. However, none of the previous reports investigated the function of MYSM1 in CRPC. Herein, we provided several lines of evidence suggesting a distinct role of MYSM1 in suppressing CRPC progression. Bioinformatics analyses of PCa cohorts from Oncomine database revealed that MYSM1 is downregulated in CRPC. In addition, the expression of MYSM1 inversely correlates with Gleason grade, Gleason score and recurrence status, establishing MYSM1 as a cancer-related marker in prostate cancer. Zhu *et al.* observed a decrease of monoubiquitinated H2A (uH2A) levels in 35 PCa patient tissues compared to corresponding normal tissues [26]. Although MYSM1 is functionally essential for deubiquitination of uH2A, the level of uH2A is regulated by multifarious deubiquitinases and ubiquitin ligases [26, 43, 45], implying that the decrease of uH2A in PCa may result from a complex process of ubiquitination and deubiquitination. In addition to clinical correlation between MYSM1 and cancer progression, our data show that ablating MYSM1 in CRPC cells robustly promotes cell growth and suppresses cellular senescence. Consistently, xenograft study that investigated the effect of MYSM1 deficiency in 22Rv1 cells revealed a significant increase of tumor volume, suggesting an antitumorigenic role for MYSM1 in CRPC. Moreover, data mining revealed a significant correlation between MYSM1 and CDK4, CCND3, CCNE1 and RB1, thus providing evidence to support the growth-suppressive role of MYSM1.

Despite the repressive function of MYSM1 in cancer progression, our findings unveiled distinct molecular mechanisms underlying MYSM1-mediated cell growth inhibition in CRPC. PTEN loss has been implicated as one cause for castration resistance in both mice and human beings [47–49]. Presurgical treatment studies have shown that PTEN inactivation is involved in hormone refractoriness after bicalutamide monotherapy [50]. The molecular mechanisms by which PTEN deficiency can result in castration resistance are poorly understood. A reciprocal negative feedback between PI3K/Akt and AR pathways has been implicated to be responsible for castration-resistant phenotype displayed in PTEN-deficient prostate cancers [21, 22]. MYSM1 has been previously demonstrated to participate in AR transcriptional activity in PTEN null LNCaP cells [26]. Importantly, we found MYSM1 knockdown has no effect on AR expression, suggesting that MYSM1 does not mediate AR target gene transcription via stabilizing AR protein. The transcriptional action of AR can be modulated by cofactors through histone modification, chromatin remodeling or manipulating the interplay between AR and transcription complex [51, 52]. Interestingly, MYSM1-associated gene LCOR has been established to act as a corepressor of nuclear hormone receptors and inhibit tumor growth and hepatic lipogenesis via physically interacting with liganded receptors [36, 53]. However, whether MYSM1 also function as a AR coregulator is not known. In this work we observed MYSM1-AR interactions which may be also responsible for the role of MYSM1 in androgen-responsive gene transcription. Although the transcriptional activity and localization of AR can be regulated by its ubiquitination status, MYSM1 functions as a histone deubiquitinase that is specific for monoubiquitinated H2A. Forming a protein complex with AR may contribute to the recruitment of MYSM1 to AR responsive elements. The localization of MYSM1 facilitates alterations of histone modification by coordinating H2A deubiquitination and histone acetylation, thus leading to transcriptional activation of AR target genes [26].

Given that MYSM1-mediated AR action is ligand-dependent, downregulation of MYSM1, in combination with androgen deprivation, dramatically impairs AR signaling in CRPC. Additionally, our findings demonstrated that MYSM1 expression is positively correlated with the levels of PTEN and PHLLPP1 both of which are well-established negative regulators of Akt signaling. Further investigation in PTEN-deficient C4-2 cells validated that inhibition of MYSM1 leads to a significant decrease in PHLPP1 expression. These observations collectively support a link between MYSM1 and PI3K/Akt pathway. Repressed AR activity resulting from MYSM1 decrease and castration therapy alleviates inhibitory feedback to Akt signaling. In agreement with the bidirectional crosstalk between PI3K/Akt and AR pathways, our data show that knockdown of MYSM1 results in increased Akt activation, which may potentially account for the malignant growth and development of antiandrogen resistance in a setting of castration.

In summary, our results unveil a pivotal role of MYSM1 in CRPC. Decreased MYSM1 may contribute to androgen-independent growth and castration resistance through modulating the reciprocal negative feedback between PI3K/Akt and AR pathways. These findings suggest a potential role of therapeutic targets and biomarkers for MYSM1 in castration-resistant prostate cancer.

## MATERIALS AND METHODS

### Prostate cancer samples

Paraffin-embedded human prostate cancer specimens were obtained from patients (n = 15) who had undergone radical prostatectomy and benign prostatic hyperplasia (BPH) specimens (n = 13) were collected by transurethral resection of prostate in Tangdu Hospital affiliated to Fourth Military Medical University (FMMU), with written informed consents from all patients. Collected samples were pathologically evaluated. Details of PCa patients were described in Supplementary Table 1. All experiments were approved by the Medical Ethics Committee of Tangdu Hospital (TDLL-201504-12).

### Cell culture

The CRPC cell lines C4-2 and 22Rv1 were obtained from American Type Culture Collection (ATCC). All cell lines were grown in Roswell Park Memorial Institute medium-1640 (RPMI-1640, Gibco) with 10% charcoal/dextran-treated FBS (Bioind) and 1% penicillin-streptomycin (Gibco). All cells were incubated at 37 °C in humidified 5% CO_2_ atmosphere.

### Oligonucleotides transfection and lentiviral transduction

All synthetic small interfering RNA (siRNA) against MYSM1 (siMYSM1) or negative control (NC) were purchased from GenePharma (Shanghai, China). Transfection of siRNA duplexes (50 nM) was carried out using Lipofectamine 2000 reagent (Invitrogen) according to the manufacturer's instructions. Lentiviral particles expressing short hairpin RNA (shRNA) targeting MYSM1 (shMYSM1) or negative control (shNC) were ordered from Obio Technology (Shanghai, China). Cells were transduced with lentiviral particles and selected with medium containing 2 μg/mL puromycin (Sigma-Aldrich) for 1 week to establish stable transfections. The detailed sequences for siRNAs and shRNAs are provided in Supplementary Table 2.

### RNA isolation and quantitative PCR (qPCR)

Total RNA was extracted from cells used in this study with TRIzol reagent (Invitrogen) following the manufacturer’s instructions. The RNA samples were subjected to reverse transcription reactions using PrimeScript™ RT Master Mix (TakaRa). Quantitative real-time polymerase chain reaction (qRT-PCR) analyses were then performed using SYBR^®^ Premix ExTaq™ II (TaKaRa) to determine the expression levels of resulting cDNA based on the manufacturer’s protocols. Beta-actin was used for an internal control. The relative abundance of mRNA was calculated via the comparative C_t_ method after normalization. The primer pairs used for qRT-PCR analyses are listed in Supplementary Table 3.

### Western blotting

Proteins for Immunoblotting were extracted using RIPA lysis buffer containing protease and phosphatase inhibitors. The concentrations of extracted proteins were quantified using BCA assay (Thermo). Reduced proteins were separated by SDS/PAGE gel and then transferred to polyvinylidene fluoride (PVDF) membrane (Millipore). After blocked in 5% bovine serum albumin (BSA) for 1h at room temperature, the PVDF membrane was incubated with indicated primary antibody overnight at 4 °C. HRP-conjugated secondary antibodies (Jackson ImmunoResearch) and ECL substrate (Millipore) were used for signal detection on FluorChem FC2 system (Alpha Innotech, San Leandro, USA) according to the manufacturer’s instructions. Detailed information of the antibodies is provided in Supplementary Table 4.

### Cell proliferation assay

Cell proliferation was measured using MTT assay kit (Sigma-Aldrich) according to instructions of the manufacturer. Briefly, Cells were seeded in triplicate at 1 × 10^3^/well in 96-well plates. Assay was performed for indicated time point by adding 20μL MTT solution to the medium. Then the plate was incubated at 37 °C. After incubation for 4h, dimethyl sulfoxide (DMSO) was used to dissolve the formazan crystals, followed by reading absorbance at 490 nm with a microplate reader (Bio-Rad). Each experiment was repeated at least three times.

### Cell cycle and apoptosis analyses

For cell cycle analysis, cells were harvested and fixed in 70% ethanol overnight at 4 °C. Then cells were treated with staining solution containing RNase A (100 μg/mL) and propidium iodide (PI, 50 μg/mL) at 37 °C for 1 h and subjected to DNA content analysis using FACS scan flow cytometer (BD Biosciences). For apoptosis analysis, cells were treated with siRNA transfection and collected for apoptosis assay at 48h after treatment. Apoptotic cells were labeled with Annexin V-FITC and PI (BD Biosciences) and quantified by CYTOMICS FC 500 flow cytometer (Beckman Coulter).

### Senescence-associated β-galactosidase (SA-β-gal) staining assay

Cellular senescence was assessed through detecting the activity of β-galactosidase using a SA-β-gal staining kit (Beyotime) according to the manufacturer’s instructions. Briefly, cultured cells in 6-well plates were immersed in fixative solution for 15 minutes at room temperature. After rinse with PBS, cells were incubated with freshly prepared staining work solution overnight at 37 °C. Then, stained cells were photographed and counted using a light microscope.

### *In vivo* tumorigenicity assay

The animal studies were approved by Institutional Animal Care and Use Committee of FMMU and conducted in accordance with ethical regulations and humane treatment. To evaluate the tumorigenicity *in vivo*, male BALB/c nude mice (6-week-old, obtained from the Experimental Animal Center of FMMU, n = 3/group) were inoculated subcutaneously with 5 × 10^6^ cells (22Rv1/shMYSM1 or 22Rv1/shNC) suspended in 100μL PBS. Tumor size was monitored every 6 days and the volume was estimated using calipers (volume = length × width^2^/2). Thirty days after inoculation, mice were sacrificed. The tumors were weighted and subjected to histological analysis.

### Immunohistochemistry (IHC)

The formalin-fixed paraffin-embedded (FFPE) sections (5 μm) were stained with hematoxylin–eosin for tumor morphology visualization. For IHC staining, xylene-deparaffinized and rehydrated sections were subjected to heat-mediated antigen retrieval with microwave in citrate buffer (pH 6.0) for 30 min. After inactivation of endogenous peroxidases (10 min, 3% H_2_O_2_) and blocking of nonspecific binding (1 h, Beyotime Immunol Staining Blocking Buffer), the sections were then incubated with diluted primary antibodies overnight at 4 °C, followed by sequential incubation with biotinylated secondary antibody (10 min, Maxim, Fuzhou, China) and streptavidin horseradish peroxidase (10 min, HRP, Maxim) at room temperature. Antigen binding was visualized using standard DAB (Maxim) staining and haematoxylin counterstaining. Images were taken under a light microscope. Detailed information of primary antibodies is provided in Supplementary Table 4.

### Co-immunoprecipitation (Co-IP) assay

Cells were lysed with IP buffer (1% NP-40, 20 mM Tris-HCl, pH 7.4, 1 mM EDTA, 150 mM NaCl, 0.25% sodium deoxycholate and protease inhibitor cocktail). Cellular extracts (1 mg) were pre-cleared with Protein A/G PLUS-Agarose beads (sc-2003, Santa Cruz Biotechnology), followed by overnight incubation with 3 μg of anti-IgG, MYSM1 or AR antibody on a rotator at 4 °C. Then Protein A/G PLUS-Agarose beads were added for 2 h incubation to precipitate immune complexes. The beads were washed five times with IP buffer and boiled for 5 min in protein loading buffer. Finally, the immunoprecipitates were subjected to Western blot analysis. Detailed information of the antibodies is provided in Supplementary Table 4.

### Bioinformatics analysis

The expression levels and clinical significance of MYSM1 in prostate cancer and other tumors were analyzed by Gene Expression Profiling Interactive Analysis (GEPIA, http://gepia.cancer-pku.cn/index.html) [54] and Oncomine (http://www.oncomine.org/). Identification of MYSM1-associated genes and gene expression correlation analysis was performed via the LinkedOmics database (http://www.linkedomics.org/) [55]. Genomic associations and network analyses of MYSM1 were carried out using Cancer Regulome Datasets (http://explorer.cancerregulome.org/). The Web-based Gene SeT AnaLysis Toolkit (WebGestalt, http://www.webgestalt.org/) was used to perform GO and KEGG pathway analyses.

### Statistical analysis

Statistical analysis was carried out using SPSS 18.0 statistical software (SPSS, IBM Corporation). Data derived from three separate experiments are shown as mean ± standard deviation (SD) or mean ± standard error of mean (SEM). Statistical analysis of two independent groups was determined by Student’s *t*-test (two-sided), except for gene expression correlation analysis that employed a Pearson correlation coefficient. For comparison of more than two groups, one-way ANOVA test was used. Survival analyses were performed by Kaplan-Meier survival curve and determined using the Log-rank test. Statistically significant differences (* *P* < 0.05, ** *P* < 0.01, *** *P* < 0.001 and **** *P* < 0.0001) were indicated.

## Supplementary Material

Supplementary Figures

Supplementary Tables
