# Supplementary material for: MYSM1-AR complex-mediated repression of Akt/c-Raf/GSK-3β signaling impedes castration-resistant prostate cancer growth
Source: Aging (Albany NY). 2019 Nov 24;11(22):10644–63. doi: 10.18632/aging.102482 (PMC6914400; doi:10.18632/aging.102482)
Supplement: Supplementary Figures [file aging-11-102482-s002..pdf]

SUPPLEMENTARY FIGURES

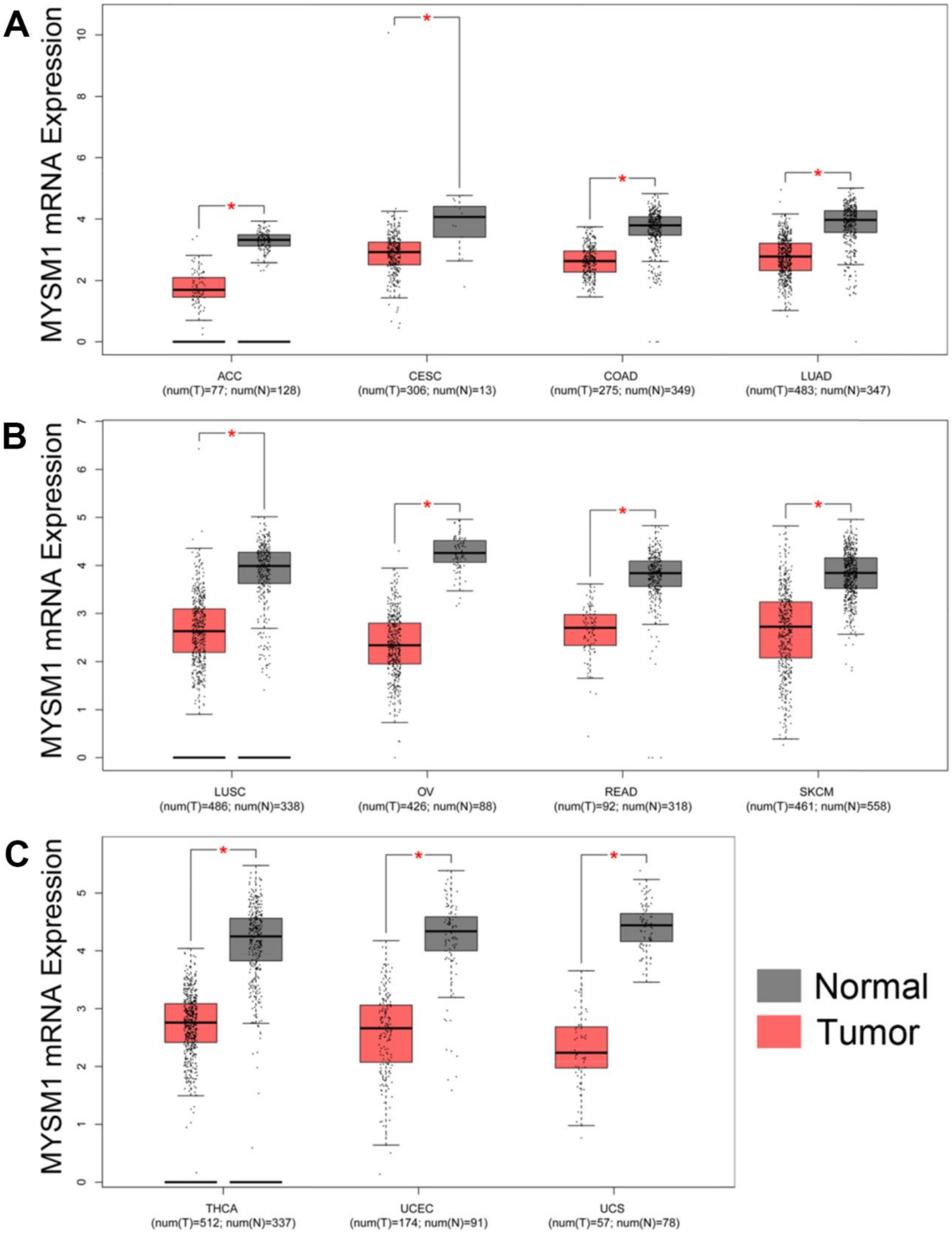

Supplementary Figure 1. MYSM1 is downregulated in numerous human tumors, including ACC, CESC, COAD, LUAD (**A**), LUSC, OV, READ, SKCM (**B**), THCA, UCEC and UCS (**C**). Data were acquired from TCGA database and analyzed via GEPIA bioinformatics.

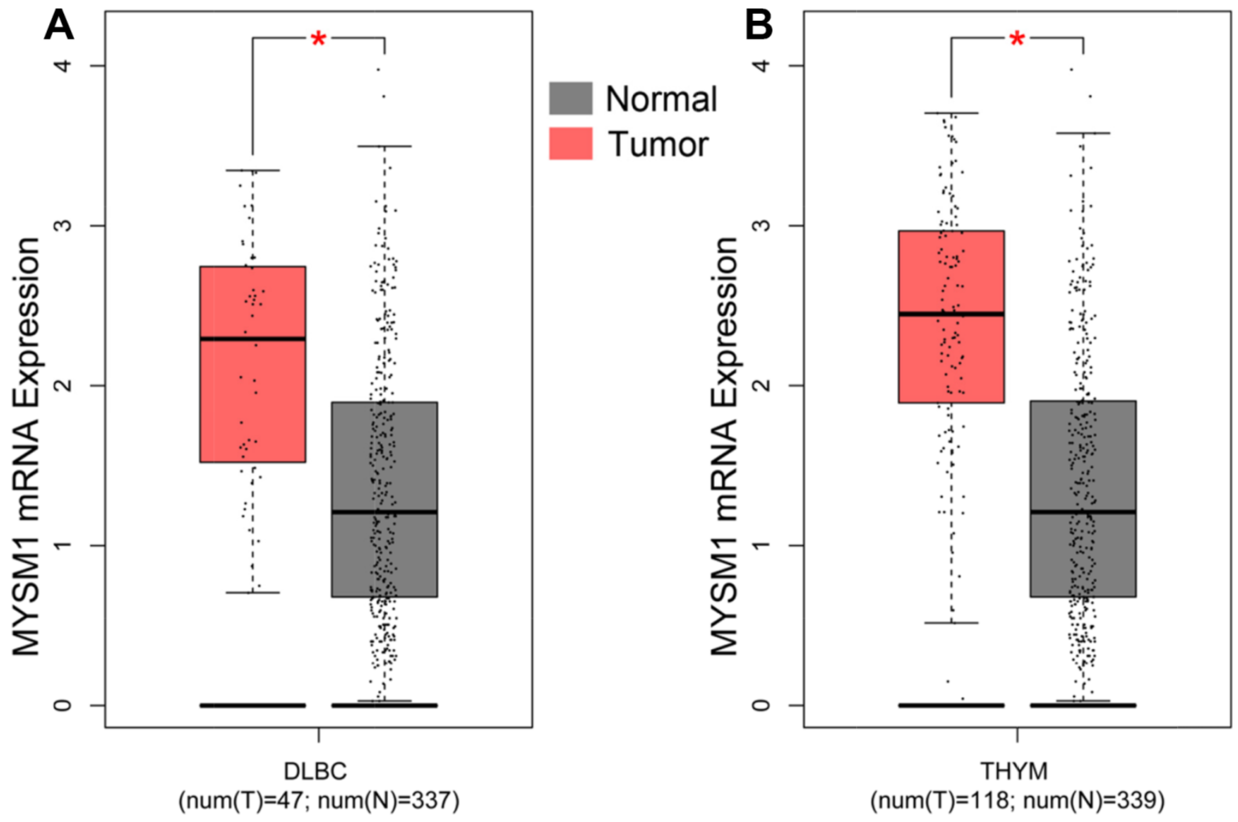

Supplementary Figure 2. Upregulated MYSM1 in DLBC (**A**) and THYM (**B**). Data were acquired from TCGA database and analyzed via GEPIA bioinformatics.

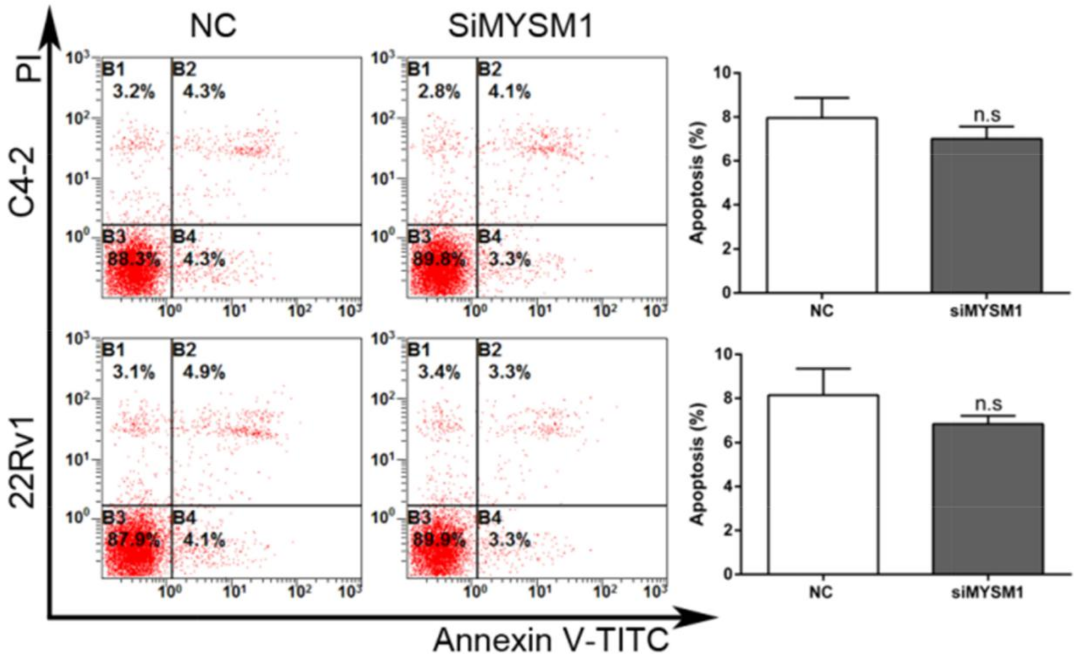

Supplementary Figure 3. Flow cytometry analysis of cell apoptosis in C4-2/22Rv1 cells treated with NC/siMYSM1. Data are shown as mean ± SD of 3 replicates. n.s = no significance.
