# Supplementary material for: MYSM1-AR complex-mediated repression of Akt/c-Raf/GSK-3β signaling impedes castration-resistant prostate cancer growth
Source: Aging (Albany NY). 2019 Nov 24;11(22):10644–63. doi: 10.18632/aging.102482 (PMC6914400; doi:10.18632/aging.102482)
Supplement: Supplementary Tables [file aging-11-102482-s001..pdf]

SUPPLEMENTARY TABLES

Supplementary Table 1. Information on PCa patient tissues collected for the study.

Patient number	ID Number	Gender	Age	PSA (ng/ml)	Gleason score
1	14876718	Male	72	24.74	5+3
2	14718613	Male	67	41.47	4+3
3	14647229	Male	71	24.99	4+3
4	14644672	Male	65	31.97	4+3
5	14483052	Male	65	13.96	3+3
6	14398145	Male	82	0.754	3+4
7	13624506	Male	66	25.18	4+3
8	11919601	Male	71	14.7	3+4
9	14931329	Male	53	43.25	4+3
10	14107451	Male	74	38.7	4+3
11	14076409	Male	70	13.41	3+4
12	14845766	Male	67	9.77	4+3
13	14233999	Male	72	19.46	5+4

Supplementary Table 2. Sequences of the oligonucleotides in this study.

Oligonucleotides	Sense (5′-3′)/Target sequence	Antisense (5′-3′)
siNC	UUCUCCGAACGUGUCACGUTT	ACGUGACACGUUCGGAGAATT
siMYSM1	CCGGCCAUAAUCUUCAAGUTT	ACUUGAAGAUUAUGGCCGGTT
shNC	TTCTCCGAACGTGTCACGT	
shMYSM1#1	CCAATCAAGGAGAATTCAT	
shMYSM1#2	CCAGAACAGGAAATAGAAA	

Supplementary Table 3. Primers used for qRT-PCR analyses in this study.

Gene	Forward (5′-3′)	Reverse (5′-3′)
MYSM1	CACAGGTACCCACATTGCTG	CTGTATCATAGGCCCCCTCA
AR	ATTGCGAGAGAGCTGCATCA	GGGCACTTGCACAGAGATGA
PHLPP1	GCAGGAAAACCTCACAGCA	AGGATGACTTGGCGTCTTGT
β-actin	TGGCATCCACGAAACTACC	GTGTTGGCGTACAGGTCTT

Supplementary Table 4. Antibodies used for Western blot analysis, Co-immunoprecipitation and Immunohistochemistry in this study.

Antigen	Species	Applications and dilutions	Source
IgG	Rabbit	3μg in Co-IP	Cell Signaling Technology #2729
MYSM1	Rabbit	WB (1:1000); 3μg in Co-IP	Abcam # ab193081
MYSM1	Rabbit	IHC (1:60)	Sangon Biotech #D261010
AR	Rabbit	WB (1:1000); IHC (1:250); 3μg in Co-IP	Abcam # ab108341
Akt(pan)	Rabbit	WB (1:1000)	Cell Signaling Technology #4691
p-Akt(Ser473)	Rabbit	WB (1:1000); IHC (1:100)	Cell Signaling Technology #4060
p-Akt(Thr308)	Rabbit	WB (1:1000)	Cell Signaling Technology #13038
c-Raf	Rabbit	WB (1:1000)	Cell Signaling Technology #9422
p-c-Raf(Ser259)	Rabbit	WB (1:1000)	Cell Signaling Technology #9421
GSK-3β	Rabbit	WB (1:1000)	Cell Signaling Technology #9315
p-GSK-3β(Ser9)	Rabbit	WB (1:1000)	Cell Signaling Technology #5558
p-PDK1(Ser241)	Rabbit	WB (1:1000)	Cell Signaling Technology #3438
Ki-67	Mouse	IHC (1:400)	Cell Signaling Technology #9449
β-actin	Mouse	WB (1:2000)	Sigma-Aldrich A1978
Anti-rabbit IgG	Goat	WB (1:5000)	Jackson ImmunoResearch #124791
Anti-mouse IgG	Goat	WB (1:5000)	Genshare Biological #JC-PB002H
